# Rare perianal extramammary Paget disease successfully treated using topical Imiquimod therapy

**DOI:** 10.1186/s12885-018-4815-6

**Published:** 2018-09-25

**Authors:** Jéssica Silva dos Santos, Gabriel Alves Bonafé, José Aires Pereira, Danilo Toshio Kanno, Carlos Augusto Real Martinez, Manoela Marques Ortega

**Affiliations:** 1 Laboratory of Cell and Molecular Tumor Biology and Bioactive Compounds, Department of Postgraduate Program in Health Science, São Francisco University (USF), Avenida São Francisco de Assis, 218, Jardim São José , Bragança Paulista, São Paulo, 12916-900 Brazil; 2 Department of Surgery and Proctology, São Francisco University (USF), Bragança Paulista, SP Brazil

**Keywords:** Perianal Paget’s disease, Histological markers, Differential diagnosis, Topical Imiquimod therapy

## Abstract

**Background:**

Perianal Paget’s disease (PPD) is a rare intraepithelial adenocarcinoma of the anal margin. Primary PPD likely represents intra-epithelial neoplasm from an apocrine source, whereas secondary disease may represent “pagetoid” spread from an anorectal malignancy.

**Case presentation:**

Histologic CDX-2 and CK20 are hallmark markers for colorectal-derived Paget’s cells. Interestingly, our primary PPD patient presented both positive and no internal malignancy was identified. In addition, a negative CK7 marker was observed in our case in contrast with previously reported. Surgical excision is the standard treatment; however, previous studies have demonstrated good response with Imiquimod 5% cream in patients with vulval extramammary Paget disease (EMPD). The efficiency of Imiquimod treatment for PPD has not been well described. Our PPD patient was successfully treated using Imiquimod 5% cream.

**Conclusions:**

This study describes a primary cutaneous PPD patient CDX-2+/CK20+/CK7- without invasion of the dermis and no associated colorectal carcinoma effectively treated using topical Imiquimod therapy, suggesting that Imiquimod might potentially be considered as a first-line treatment for PPD.

## Background

Paget’s disease, described by Sir James Paget in 1874 [1], is classified as mammary and extramammary subgroups. Extramammary Paget’s disease (EMPD) is a rare condition that is part of a spectrum of intraepithelial adenocarcinomas characterized by proliferation of apocrine cells called Paget cells, located in the basal layer of the skin and often can reach to the entire epithelium thickness [2]. EMPD incidence age is 50 to 80 with the peak age of incidence being 65 years [2]. EMPD of the vulva is most common accounting for up to 65% of cases while other areas affected include perianal area, male groin, scrotum, and axillae [2]. Perianal Paget’s disease (PPD) is a subgroup of EMPD, which is an uncommon intraepithelial neoplasm. The true incidence of the disease is difficult to estimate due to its rarity, but it is known to represent less than 1% of all anal diseases and 6.5% of all cases of Paget’s disease [3]. Although its etiology has never been definitively determined, there are two types of PPD, primary likely represents intra-epithelial neoplasm from an apocrine source, whereas secondary disease may represent “pagetoid” spread from an anorectal malignancy [4].

In Brazil, Tanaka et al. [5] reported 14 cases of EMPD, four of which involved women with disease in the vulvar, groin or perianal areas. Trindade et al. [6] reported a primary Brazilian PPD patient, which presented erythematous, itching and little bleeding. The staging tests were normal, without evidence of metastasis.

Imiquimod is an immune response modulating drug that targets toll-like receptors of dendritic and Langerhans cells, which results in the release of multiple cytokines and can directly induce apoptosis of transformed epithelial cells [7]. Imiquimod is a drug of choice for genital warts, vulvar and vaginal intraepithelial neoplasia, and actinic keratosis [8], and it has emerged as a promising drug for the treatment of vulvar and scrotal EMPD [9–11]. However, the exact statistics for PPD treatment efficiency with Imiquimod have not been well described.

The aim of this study was to describe a case of PPD in Brazil, and the effectiveness of topical Imiquimod therapy.

## Case presentation

This study was conducted according to the Declaration of Helsinki, and was approved by the local institutional review board guidelines (number: 54306316.4.0000.5514).

**Fig. 1:**
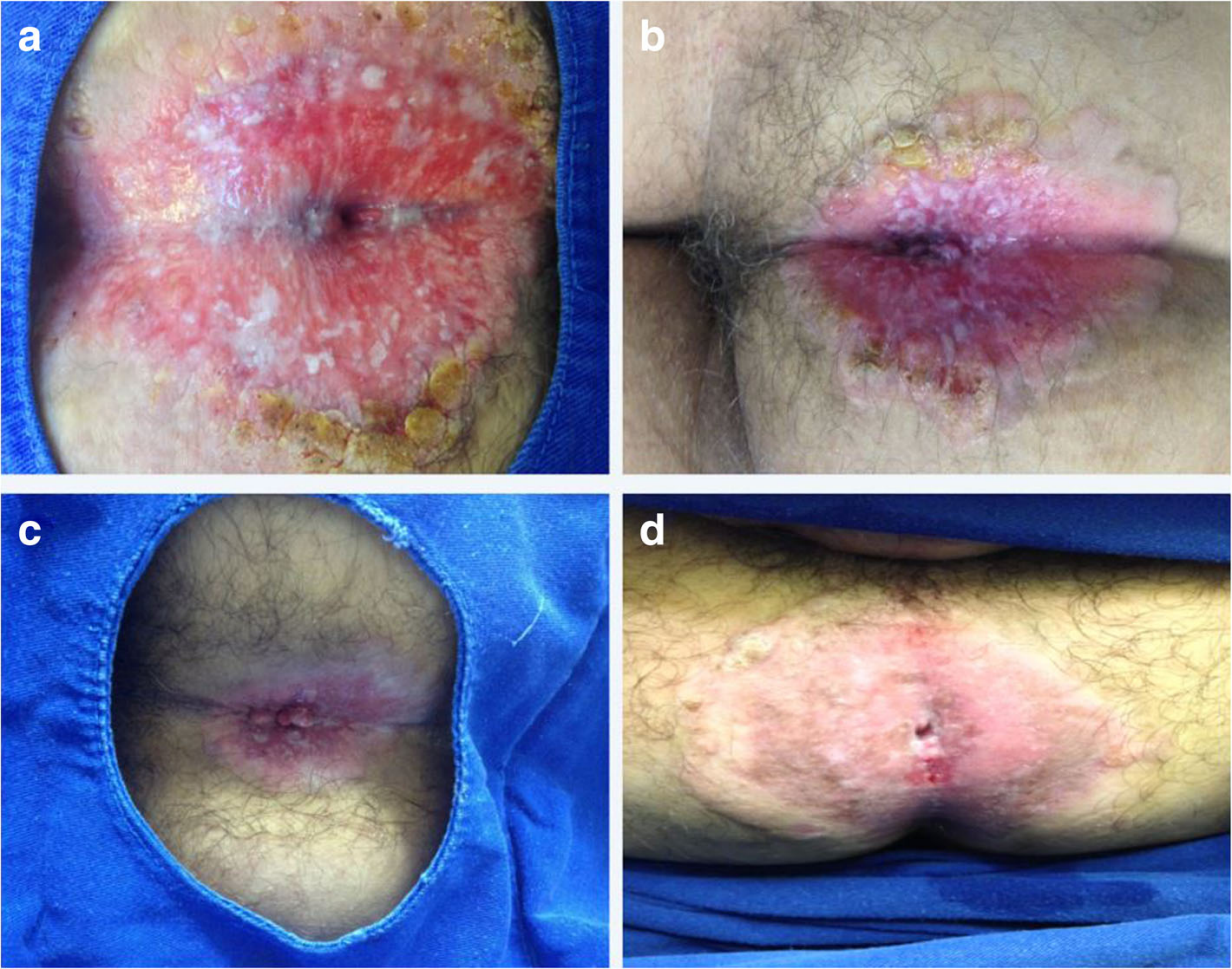
Colonoscopy examination in the perianal Paget’s disease (PPD) patient revealed skin condition improvement (**a**) Presence of intense perianal rash with xeroderma, peeling skin, warty lesions and a severe stenosis of the anal orifice. **b-d** After 4, 8 and 12 months treatment using Imiquimod 5% cream, respectively

The patient, a 66-year-old Brazilian male sought the Coloproctology Division at USF, Bragança Paulista, São Paulo, Brazil due to a complaint of an anal lump for 3 years, and fistula with absolute hyperemia and decreased caliber of the stool accompanied by increased constipation with occasional rectum bleeding. The colonoscopy showed the presence of intense perianal rash with xeroderma, peeling skin, warty lesions and a severe stenosis of the anal orifice (Fig. 1a). Also, no changes in the rectal mucosa and absence of polyps in the colon were observed.

**Fig. 2:**
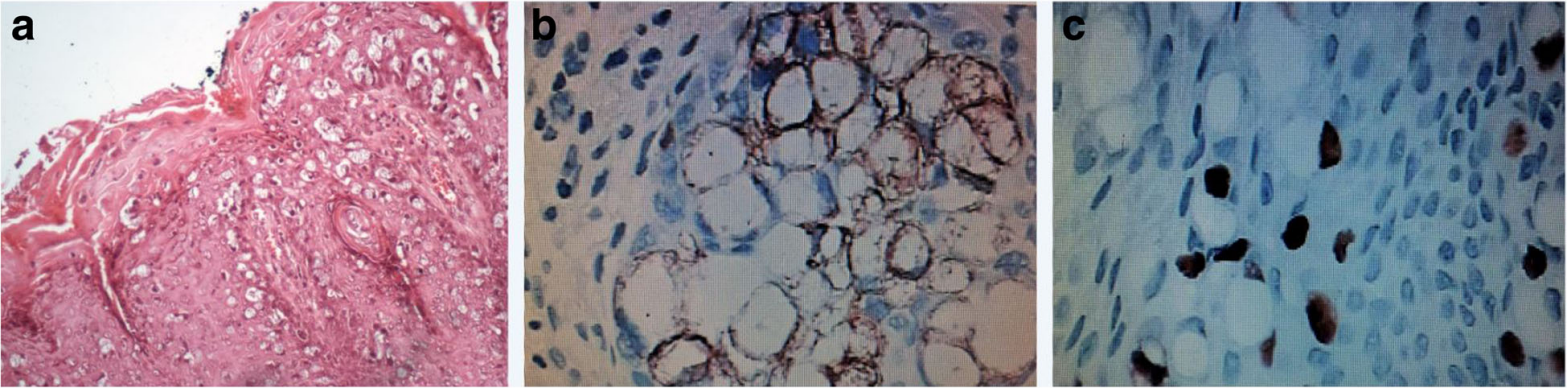
Histologic Findings of a Case of a Perianal Paget’s disease (PPD) (**a**) PPD diagnostic was obtained by histological examination showing the presence of Paget cells which are larger than the surrounding keratinocytes and have prominent nuclei and moderate amounts of pale cytoplasm (hematoxylin and eosin stain, original magnification × 200). **b** Positive immunostaining for cytokeratin 20 (CD20) (original magnification × 400). **c** Positive immunostaining for CDX-2 (original magnification × 400)

Perianal surgical biopsies were performed with segments of skin resection and subcutaneous tissue at areas where there was a lack of skin irregularity and hardened consistency. Large biopsies fragments of four quadrants were removed. Post-treatment biopsies were performed using the same technique. Perianal biopsy showed the atypical Paget cells suggesting PPD or balloon cells melanoma (Fig. 2a). For the differential diagnosis, Melan-A, CDX-2, cytokeratin 20 (CK20), cytokeratin 7 (CK7), p63, and human epidermal growth factor receptor 2 (HER2) immunostaining markers panel was analyzed. Positive staining only for cytokeratin 20 (Fig. 2b) and CDX-2 (Fig. 2c) markers was observed. No PPD diagnosis secondary to a colorectal origin was confirmed. The anal orifice stenosis was treated by sphincterotomy.

Local excision is considered the first-line therapy in PPD cases and most of them requiring also an abdominoperineal amputation of the rectum with a permanent colostomy. The patient refused that option. Then, since there was no dermis invasion and no associated colorectal carcinoma, the PPD treatment choice was Imiquimod 5% cream for 12 months. Every 4 months a new colonoscopy revealed skin condition improvement (Fig. 1b-d) and perianal biopsy showed no residual PPD (data not shown).

After the first Imiquimod treatment cycle, two more surgical biopsies were performed and a new Imiquimod cycle was prescribed. After 3 months a third biopsy revealed no sign of lesion. The patient is currently being closely followed every 3 months. The patient remains without signs of recurrence to date. If no lesion is observed, the follow-up may include biopsy from the old perianal lesion once a year and colonoscopy once every 2 years.

It is interestingly to note that patient has presented pain and irritation of the perianal skin during the period of Imiquimod application and the inflammatory process completely improved after the first 45 days with remaining residual local pruritus.

## Conclusions

PPD is categorized into primary cutaneous origin and secondary due to the extension of a visceral malignancy as anorectal or colon. Grow et al. [12] reported a 76% association of PPD with adjacent or bowel carcinoma. This study describes a primary cutaneous PPD patient without invasion of the dermis and no associated colorectal carcinoma.

Differential diagnoses of PPD include Bowen’s disease, contact dermatitis, lichenoid lesions, psoriasis, melanoma, perianal Crohn’s involvement, mycosis fungoides, squamous cell carcinoma and tinea cruris [13]. Immunostaining markers may enhance the diagnostic accuracy. CDX-2 and CK20 positive markers have been known as markers for colorectal-derived Paget’s cells [14–16]. Interestingly, our primary cutaneous PPD patient presented both markers positive and no internal malignancy was identified. CDX-2 sensitivity is modest and it must be analyzed in conjunction with additional immunostaining markers [17]. The negative CK7 marker was observed in our case. Most of the publications confirm that primary PPD is usually CK7 positive and CK20 negative [18, 19], whereas EMPD secondary to colorectal carcinoma is positive for CK7 and CK20 [18].

Melan-2 is the melanocyte differentiation marker, and it is negativity excludes amelanotic melanoma [20]. Overexpression of HER-2 protein is seen in 20–60% cases of EMPD and signifies poor prognosis often due to deep dermal invasion or metastatic disease [21]. The p63 expression in the epidermis and sebaceous glands is useful to differentiate pagetoid bowels disease from EMPD [22]. Our PPD patient presented both HER and p63 negative confirming no internal malignancies or metastasis.

The primary PPD diagnosis must be established using histological examination showing the presence of Paget cells and different histological markers to establish primary cutaneous origin or secondary internal malignancy.

A wide range of PPD treatment modalities have been previously reported, including surgery [23, 24], abdominoperineal resection [25], radiotherapy [26], and chemo-radiotherapy [27]. Few authors have suggested nonsurgical treatment first. Kobayashi et al. [28] reported the effect of intralesional interferon. Our case was treated using Imiquimod 5% cream and complete healing without recurrence of PPD was observed. One previous report showed similar results in a long-lasting PPD patient without deep gastrointestinal neoplasia [29]. Recently, another report described the primary local treatment of PPD with Imiquimod 5% cream over a 16-week period initially resulting in remission prior to lymph node spread at 18 months [19].

The present case corresponds to stage I according to Shutze and Gleysteen classification [30]. Recommendation for stages I and II is a wide local excision [13]; however, Imiquimod was successfully an alternative treatment in our case. Long-term follow-up is required to exclude the recurrence of the disease and development of associated tumors.

In conclusion, this study reports clinical characteristics of this rare disease in a Brazilian patient and suggests the use of a nonsurgical treatment for early-stage PPD.

## Abbreviations

EMPD: Extramammary Paget disease
PPD: Perianal Paget’s disease

## References

[1] Paget J (1874). On the disease of the mammary gland areola preceding cancer of the mammary gland. St Bartholomew’s Hosp Rep.

[2] Lam Christina, Funaro Deana (2010). Extramammary Paget’s Disease: Summary of Current Knowledge. Dermatologic Clinics.

[3] Kyriazanos Ioannis D., Stamos Nikolaos P., Miliadis Lazaros, Noussis Grigorios, Stoidis Christos N. (2011). Extra-mammary Paget’s disease of the perianal region: A review of the literature emphasizing the operative management technique. Surgical Oncology.

[4] Park JS, Kerner BA (2003). Perianal Paget’s disease. Semin Colon Rectal Surg.

[5] Tanaka VDA, Sanches JA, Torezan L, Niwa AB, Neto CF (2009). Mammary and extramammary paget’s disease: a study of 14 cases and the associated therapeutic difficulties. Clinics (São Paulo).

[6] Trindade Etelvino de Souza, Polcheira Paulo Arlindo, Basílio Dúnya Bachour, Rocha Zali Neves da, Rocha Júnior José Lopes, Primo Guttenberg Rodrigues Pereira (2004). Doença de Paget invasiva da vulva e região perianal: relato de caso. Revista Brasileira de Ginecologia e Obstetrícia.

[7] Huang S-W., Liu K-T., Chang C-C., Chen Y-J., Wu C-Y., Tsai J-J., Lu W-C., Wang Y-T., Liu C-M., Shieh J-J. (2010). Imiquimod simultaneously induces autophagy and apoptosis in human basal cell carcinoma cells. British Journal of Dermatology.

[8] Kemeny L, Nagy N (2010). New perspective in immunotherapy: local imiquimod treatment. Orv Hetil.

[9] Fehres CM, Bruijns SC, van Beelen AJ, Kalay H, Ambrosini M, Hooijberg E, Unger WW, Gruijil TD, Van KY (2014). Topical rather than intradermal application of the TLR7 ligand imiquimod leads to human dermal dendritic cell maturation and CD8+ T-cell cross-priming. Eur J Immunol.

[10] Isik Ozgen, Aytac Erman, Brainard Jennifer, Valente Michael A., Abbas Maher A., Gorgun Emre (2015). Perianal Paget’s disease: three decades experience of a single institution. International Journal of Colorectal Disease.

[11] Kim TH, Chang IH, Kim TH, Lee SY, Myung SC (2009). Extramammary Paget's disease of scrotum treated with radiotherapy. Urology.

[12] Grow JR, Kshirsagar V, Tolentino M (1997). Extramammary perianal Paget’s disease: a report of a case. Dis Colon Rectum.

[13] Jankulovski N, Spasevska L, Janevska V, Dukova B (2013). A true epidermotropic apocrine neoplasm in the form of perianal Paget’s disease: a case report. J Med Case Rep.

[14] De Nisi MC, D’Amuri A, Toscano M, Lalinga AV, Pirtoli L, Miracco C. Usefulness of CDX2 in the diagnosis of extramammary Paget disease associated with malignancies of intestinal type. Br J Dermatol. 2005;(3):677–9. 10.1111/j.1365-2133.2005.06798.x 16120171

[15] Zeng Hao Audrey, Cartun Richard, Ricci Andrew (2005). Potential Diagnostic Utility of CDX-2 Immunophenotyping in Extramammary Paget??s Disease. Applied Immunohistochemistry & Molecular Morphology.

[16] Ramalingam P, Hart WR, Goldblum JR (2001). Cytokeratin subset immunostaining in rectal adenocarcinoma and normal anal glands. Arch Pathol Lab Med.

[17] Lora V, Kanitakis J (2009). CDX2 expression in cutaneous metastatic carcinomas and extramammary Paget’s disease. Anticancer Res.

[18] Le Fur R, Mears L, Dannawi Z. A perianal extramammary Paget’s disease associated with two well-differentiated invasive intramucosal sigmoid carcinomas, a very rare case: an immunohistochemical and clinical review of extramammary Paget’s disease. Ann R Coll Surg Engl 2004; 6:w26–w31. 10.1308/147870804876 PMC1964294 16749961

[19] Knight Stephen R., Proby Charlotte, Ziyaie Dorin, Carey Frank, Koch Sacha (2016). Extramammary Paget disease of the perianal region: the potential role of imiquimod in achieving disease control. Journal of Surgical Case Reports.

[20] Kaufmann O, Koch S, Burghardt J, Audring H, Dietel M (1998). Tyrosinase, melan-a, and KBA62 as markers for the immunohistochemical identification of metastatic amelanotic melanomas on paraffin sections. Mod Pathol.

[21] TAKAHAGI Shunsuke, NODA Hideki, KAMEGASHIRA Akiko, MADOKORO Naoki, HORI Ikuko, SHINDO Hajime, MIHARA Shouji, HIDE Michihiro (2009). Metastatic extramammary Paget’s disease treated with paclitaxel and trastuzumab combination chemotherapy. The Journal of Dermatology.

[22] Memezawa A, Okuyama R, Tagami H, Aiba S (2008). P63 constitutes a useful histochemical marker for differentiation of Pagetoid Bowen’s disease from Extramammary Paget’s disease. Acta Derm Venereol.

[23] Murakami Koichi, Tanimura Hiroshi, Ishimoto Kiwao, Yamaue Hiroki, Yamade Naohisa, Shimamoto Tetsuya (1996). Reconstruction with bilateral gluteus maximus myocutaneous rotation flap after wide local excision for perianal extramammary Pagetʼs disease. Diseases of the Colon & Rectum.

[24] Lam David T. Y., Batista Oscar, Weiss Eric G., Nogueras Juan J., Wexner Steven D. (2001). Staged excision and split-thickness skin graft for circumferential perianal Pagetʼs disease. Diseases of the Colon & Rectum.

[25] McCarter Martin D., Quan Stuart H. Q., Busam Klaus, Paty Philip P., Wong Douglas, Guillem Jose G. (2003). Long-Term Outcome of Perianal Pagetʼs Disease. Diseases of the Colon & Rectum.

[26] Brown RS, Lankester KJ, McCormack M. Radiotherapy for perineal Paget’s disease. Clin Oncol. 2002;(4):272–84. 10.1053/clon.2002.0092 12206637

[27] Thirlby RC, Hammer CJ Jr, Galagan KA, Travaglini JJ, Picozzi VJ Jr. Perianal Paget's disease: successful treatment with combined chemoradiotherapy. Report of a case. Dis Colon Rectum. 1990;(2):150–2. 10.1007/BF02055547 2153511

[28] Kobayashi H, Someda Y, Furukawa M, Chanoki M, Hamada T (1987). Intralesional interferon in the treatment of extramammary Paget’s disease. Nihon Hifuka Gakkai Zasshi.

[29] Vereecken P, Awada A, Ghanem G, Marques da Costa C, Larsimont D, Simoens C, Mendes CP, Hendkisz U (2007). A therapeutic approach to perianal extramammary Paget's disease: topical imiquimod can be useful to prevent or defer surgery. Med Sci Monit.

[30] Shutze WP, Gleysteen JJ. Perianal Paget's disease. Classification and review of management: report of two cases. Dis Colon Rectum. 1990;(6):502–7. 10.1007/BF02052147 2161727

